# Cost-effectiveness of the Adaptive Implementation of Effective Programs Trial (ADEPT): approaches to adopting implementation strategies

**DOI:** 10.1186/s13012-020-01069-w

**Published:** 2020-12-14

**Authors:** Andria B. Eisman, David W. Hutton, Lisa A. Prosser, Shawna N. Smith, Amy M. Kilbourne

**Affiliations:** 1 Community Health, Division of Kinesiology, Health and Sport Studies, College of Education, Wayne State University, 2153 Faculty/Administration Building, 656 West Kirby, Detroit, MI 48202 USA; 2 Department of Health Management and Policy, School of Public Health, University of Michigan, Ann Arbor, MI 48109 USA; 3 Susan B. Meister Child Health Evaluation & Research Center, Dept of Pediatrics, University of Michigan Medical School, Ann Arbor, MI 48109 USA; 4 Department of Psychiatry, University of Michigan Medical School, Ann Arbor, MI 48109 USA; 5 Department of Learning Health Sciences, University of Michigan Medical School, Ann Arbor, MI USA; 6 Quality Enhancement Research Initiative, U.S. Department of Veterans Affairs, Washington, USA

**Keywords:** Implementation science, Costs and cost analysis, Mental health services, community, Cost-effectiveness analysis

## Abstract

**Background:**

Theory-based methods to support the uptake of evidence-based practices (EBPs) are critical to improving mental health outcomes. Implementation strategy costs can be substantial, and few have been rigorously evaluated. The purpose of this study is to conduct a cost-effectiveness analysis to identify the most cost-effective approach to deploying implementation strategies to enhance the uptake of Life Goals, a mental health EBP.

**Methods:**

We used data from a previously conducted randomized trial to compare the cost-effectiveness of Replicating Effective Programs (REP) combined with external and/or internal facilitation among sites non-responsive to REP. REP is a low-level strategy that includes EBP packaging, training, and technical assistance. External facilitation (EF) involves external expert support, and internal facilitation (IF) augments EF with protected time for internal staff to support EBP implementation. We developed a decision tree to assess 1-year costs and outcomes for four implementation strategies: (1) REP only, (2) REP+EF, (3) REP+EF add IF if needed, (4) REP+EF/IF. The analysis used a 1-year time horizon and assumed a health payer perspective. Our outcome was quality-adjusted life years (QALYs). The economic outcome was the incremental cost-effectiveness ratio (ICER). We conducted deterministic and probabilistic sensitivity analysis (PSA).

**Results:**

Our results indicate that REP+EF add IF is the most cost-effective option with an ICER of $593/QALY. The REP+EF/IF and REP+EF only conditions are dominated (i.e., more expensive and less effective than comparators). One-way sensitivity analyses indicate that results are sensitive to utilities for REP+EF and REP+EF add IF. The PSA results indicate that REP+EF, add IF is the optimal strategy in 30% of iterations at the threshold of $100,000/QALY.

**Conclusions:**

Our results suggest that the most cost-effective implementation support begins with a less intensive, less costly strategy initially and increases as needed to enhance EBP uptake. Using this approach, implementation support resources can be judiciously allocated to those clinics that would most benefit. Our results were not robust to changes in the utility measure. Research is needed that incorporates robust and relevant utilities in implementation studies to determine the most cost-effective strategies. This study advances economic evaluation of implementation by assessing costs and utilities across multiple implementation strategy combinations.

**Trial registration:**

ClinicalTrials.gov Identifier: NCT02151331, 05/30/2014.

Contributions to the literature

- Researchers to date have focused primarily on quantifying intervention costs; few have focused on implementation strategy costs and cost-effectiveness.
- This research focuses on advancing approaches for evaluating cost and cost-effectiveness of implementation strategies, which are provider tools/strategies to promote intervention uptake and have been understudied.
- This study is one of the first to conduct a comparative economic analysis of an adaptive implementation strategy trial, to provide useful, accessible information for communities to make well-informed decisions about resourcing implementation investments.

## Background

Evidence-based treatments for mental health conditions, including depression, are essential to improving the public’s health [1]. Mental health conditions frequently co-occur with substance use disorders, and other co-occurring conditions, inciting sequelae of short- and long-term consequences [2]. Mental health conditions have a significant financial toll: researchers estimated in 2008 that the annual earnings loss for serious mental illness in 2008 was $193.2 billion [3]. Collaborative care models (CCMs) have demonstrated effectiveness in improving outcomes among patients with mental disorders; collaborative care models such as Life Goals are designed to improve medical *and* psychiatric outcomes for persons with mood disorders through personal goal-setting aligned with wellness and symptom coping strategies and supported through collaborative care [4–6]. Life Goals is an evidence-based CCM that focuses on three components recognized as central to effective CCMs: patient self-management, care management, and provider decision support [7, 8]. Several randomized trials have shown Life Goals to be effective in improving mental and physical health outcomes for patients with unipolar and bipolar depression [4–6, 9]. The Life Goals self-management component comprises six psychosocial sessions for patients, to be delivered in either individual or group format. While all Life Goals patients complete core introduction and conclusion modules, the four intermediary sessions can be chosen by patients and providers from among several mental health and wellness subjects, including depression, mania, physical activity, or substance abuse. Life Goals also provides manualized support for care management and provider decision support, including templates for tracking patient progress and guides to common medications for unipolar/bipolar depression patients. Most individuals suffering from depression and other mental health conditions are not receiving evidence-based practices (EBPs) such as Life Goals in community settings, resulting in poor and costly health outcomes and millions of research dollars wasted when EBPs fail to reach those most in need [10–12]. Researchers increasingly recognize that EBPs must be complemented by effective implementation strategies (i.e., implementation interventions) to achieve desired public health outcomes [13]. Replicating Effective Programs (REP) is an implementation strategy focused on maximizing flexibility *and* fidelity in EBP delivery [14]. REP, based on the CDC’s research-to-practice framework [15], is guided by Social Learning [16] and Diffusion of Innovations Theories [17]*.* Standard REP includes three primary components: program packaging, provider training, and facilitation. Standard REP is a low intensity, minimal cost intervention that is akin to standard implementation for many evidence-based programs and practices; standard REP has improved uptake of brief HIV-focused interventions but has been less successful with the uptake of more complex behavioral interventions [18]. Researchers have also developed enhanced REP for more complex clinical behavioral interventions, which include added customization for program packaging and training, and implementation facilitation [19]. Implementation facilitation (i.e., facilitation) is a promising implementation strategy from the integrating Promoting Action on Research Implementation in Health Services (iPARIHS) framework that provides ongoing, individualized assistance for program delivery that can help enhance uptake of EBPs such as Life Goals in community clinics [19, 20]. Facilitation applies principles of interactive problem solving *with* practice-based knowledge to support providers as they engage in program delivery [21, 22]. Individuals within (internal facilitator, IF) and outside of (external facilitator, EF) the organization can provide ongoing support for EBP implementation [19]. External facilitators (EF) provide expertise, active guidance, and support for intervention delivery. Internal facilitators (IF) work in tandem with EFs to support providers in program delivery and communicate with organizational leadership and the external facilitator.

The costs associated with implementation strategies, especially multicomponent strategies such as REP+facilitation, can be substantial. Cost is a key consideration from an organizational or system perspective when implementing new innovations [11]. Understanding the resources needed to achieve desired behavioral outcomes (e.g., improved mental health) is essential to implementing and sustaining EBPs in communities [23]. Most economic evaluation of implementation, however, has focused on intervention costs and *not* the costs of implementation strategies required to deploy and sustain them [24]. Economic evaluation of implementation refers to the systematic evaluation of what outcomes a specific implementation strategy or set of competing strategies achieves and the costs of achieving them [25]. Economic evaluation provides key information for decision makers regarding implementation strategies to support and sustain EBP delivery*.* Organizations benefit from evidence that supports (or refutes) investment in specific strategies as an efficient use of resources, and this can help prioritize implementation efforts [11, 24, 26]. Despite this need for practical economic information that will provide decision makers with information on whether the cost of deploying an implementation strategy is worth the added cost (versus standard implementation or an alternative strategy), less than 10% of implementation studies include cost information, and even fewer conduct comparative economic analyses [25, 27]. Thus, additional research is needed to advance economic evaluation of implementation as this will be instrumental in demonstrating if investment in implementation strategies is worth the additional costs [28].

Many types of cost evaluation exist, but one well suited to implementation science is cost-effectiveness analysis. Cost-effectiveness analysis (CEA) assesses whether incremental benefits of one strategy versus another are sufficient to justify additional costs and has been used to support mental health treatment-focused EBPs for clinical settings [29]. CEA can inform decisions about resource allocation for program selection and delivery [30].

**Fig. 1:**
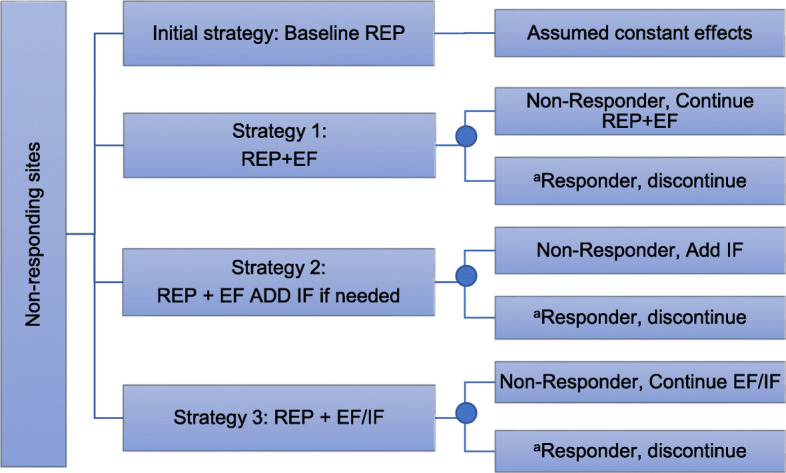
Decision tree of the ADEPT trial. ^a^Sites that responded to the implementation strategy after the initial 6 months of the Trial Phase: either < 10 patients receiving Life Goals or > 50% of patients receiving Life Goals had ≤ 3 sessions, min dose for clinically significant results. Sites that responded to the implementation strategy discontinued the strategy during the second 6 months/Phase III of the trial

The objective of this study is to estimate the costs and conduct a CEA as part of an adaptive implementation trial comparing different implementation strategies. The goal of Adaptive Implementation of Effective Programs Trial (ADEPT) is to use a sequential multiple assignment randomized trial (SMART) design to compare the effectiveness of different augmentations to REP using EF or a combination of EF + IF on mental health outcomes among patients diagnosed with depression or bipolar disorders in community-based practices; details of the ADEPT trial are described in more detail elsewhere [19]. A secondary ADEPT aim was to assess the costs for different scenarios of combining REP+facilitation (see Fig. 1 and Fig. 4 in the Appendix) to identify the most cost-effective implementation strategy approach. We compare four different implementation strategy combinations and evaluate relative cost-effectiveness to identify which implementation strategies are most cost-effective in achievi program goals: Strategy 0: REP only, Strategy 1: REP+EF, Strategy 2: REP+EF add IF if needed, and Strategy 3: REP+EF/IF. Clinics responding to their respective implementation strategy (e.g., > 10 patients receiving Life Goals) discontinued the implementation strategy. Among those that did not respond during the second phase of the trial, for the final phase Strategy 1 continued with EF, Strategy 2 added IF, and Strategy 3 continued with EF/IF.

## Methods

This study will use a simulation modeling approach using data from a previously conducted clinical trial [19, 31]. Our results are reported using the Consolidated Health Economic Evaluation Reporting (CHEERS) guidelines [32]. Implementation strategies included in the model reflect implementation strategies that could be developed using data from the trial. In this study, we focus on the ADEPT community-based mental health or primary care clinics who were non-responsive after 6 months of Replicating Effective Programs (REP) and would receive additional implementation support (i.e., facilitation) to enhance uptake of Life Goals. Non-responsive to REP was defined as 10 or fewer patients receiving Life Goals or < 50% of patients receiving a clinically significant dose of Life Goals, fewer than three Life Goals sessions (< 3 out of 6), after 6 months [33–35]. Eligible sites had at least 100 unique patients diagnosed with depression and could designate at least 1 mental health provider to administer individual or group collaborative care sessions for patients. The study was approved by local institutional review boards (IRBs) and registered under clinicaltrials.gov (identifier: NCT02151331).

### Modeling approach

Using data from the ADEPT trial, we designed a cost-effectiveness study to evaluate three strategies that could be implemented to support the uptake and clinical effectiveness of Life Goals. These strategies do not exactly match the arms in the clinical trial because our goal was to evaluate the optimal implementation strategy approach among non-responders. We developed a decision tree to assess 1-year costs and outcomes for different intervention strategies following 6 months of REP (baseline) among non-responsive sites (i.e., slow adopter sites). Implementation strategies included in the model (see Fig. 1) were as follows: Strategy 0: REP only, Strategy 1: REP+EF, Strategy 2: REP+EF, ADD IF if needed, and Strategy 3: REP+EF/IF. The probability of non-response to the implementation strategies in the model was based on observed response rates in the study, which remained consistent across each phase at approximately .09 (that is, 9% were responders). Sites who responded to their assigned implementation strategy after 6 months of the trial (Phase II) discontinued the strategy. Sites who did not respond at after 6 months proceeded with Phase III as follows: for Strategy 1: continued REP+EF, for Strategy 2, added IF and for Strategy 3 continued with REP+EF/IF. The analysis uses a 1-year time horizon and assumes a health sector perspective. Parameter inputs were derived using primary data from ADEPT.

### Costs

Implementation strategy costs for baseline REP were the same for all participants and include costs for training program providers, training compensation (e.g., pay during non-work hours), time costs for assessing organizational needs, and pre-implementation meetings. Non-labor costs included costs of the curriculum (manual and materials) and travel costs [24, 36]. Facilitation costs were based on the facilitation logs. The study EF and site IFs logged their tasks, categorizing mode, personnel interaction, duration, and the primary focus of each task. These tasks included coaching, developing an implementation plan, education, linking to outside resources, and consultation. We calculated costs based on time spent by hourly wage plus fringe rates for facilitators. As there was one EF employed by the study team, we used the EF hourly wage + fringe. For the IFs, training, and background (and thus costs) varied. We based the IF salary and fringe rates on current rates for Licensed Masters of Social Work (LMSW) professional using Bureau of Labor Statistics data, as many of the IFs were LMSWs. As we anticipated differences in uptake, that is the number of patients receiving Life Goals by condition, we calculated the total site-level cost per strategy (the level of randomization) and divided by the number of patients in that implementation strategy condition. The number of patients per condition was obtained from site-level records. Costs were collected in 2014 and adjusted to US 2018 dollars using the Consumer Price Index [37]. A summary of cost parameters is provided in Table 1. We report summary statistics for implementation costs with 95% confidence intervals. We estimated the costs of REP using the available cost data to obtain a comprehensive assessment of total implementation intervention costs, plus the costs of facilitation activities in each condition (EF and EF/IF).

**Table 1:** Model inputs

Parameter	Base	Low	High	Distribution^a^	Source
Costs					
Cost of REP (Phase I)^b^	588.95	0	558.95	Normal	Time and resource tracking, study staff
Additional cost of EF (Phase II)	32.70	32.39	33.01	Normal	Time logs
Additional cost of EF (Phase III)	17.55	1.22	30.84	Normal	Time logs
Additional cost of EF and IF (Phase II)	31.23	28.51	30.49	Normal	Time logs
Additional cost of EF and IF (Phase III)	6.35	3.15	9.27	Normal	Time logs
Probabilities					
Probability of response after Phase II with REP+EF	0.095	0	0.095		Site response data
Probability of response after Phase II with REP+EF/IF	0.091	0	0.091		Site response data
Utilities^c,d^					
REP only	.475	0.43	0.521	Beta	Patient survey
EF (Phase II)	.497	0.42	0.573	Beta	Patient survey
EF non-responding site (Phase III)	.446	0.306	0.586	Beta	Patient survey
EF responding site (Phase III)	.721	0.533	0.909	Beta	Patient survey
EF and IF (Phase II)	.463	0.362	0.564	Beta	Patient survey
EF add IF (Phase III)	.568	0.392	0.566	Beta	Patient survey
EF and IF non-responding site (Phase III)	.479	0.392	0.566	Beta	Patient survey
EF and IF responding site (Phase III)	.372	0.184	0.559	Beta	Patient survey

^a^Distributions are parameterized such that the mean is the base value and the standard deviation is ¼ of the difference between the low and high values

^b^Phases refer to values calculated within phases of the original trial: Phase I: baseline/initial 6-month period prior to the trial phase of the study with REP only, Phase II: second 6 months of the study, Phase III: final 6 months of the trial

^c^Inverse probability weighting to account for missing data with weights estimated from a logistic regression model for predicting non-response

^d^EQ-5D calculated using mapping algorithm from components of the SF-12

### Health outcomes

#### Quality-adjusted life years (QALYs)

To develop a preference-based health utility measure for the current study, we mapped the SF-12 (which was collected as part of the patient-level evaluation in the ADEPT trial) to the EQ-5D, a multi-attribute utility instrument, using an established algorithm developed by Franks and colleagues [38]. The EQ-5D yields interval-level scores ranging from 0 (dead) to 1 (perfect health). This mapping provides a health utility measure for each health state experienced by patients in the study and can be used to calculate quality-adjusted life years, the preferred measure for health benefits used in cost-effectiveness analysis.

### Data analytic approach

We used a decision-tree model to compare the cost-effectiveness across different scenarios for combining REP+facilitation for the Life Goals EBP (see Fig. 1 and Fig. 4 in the Appendix). The time horizon for this analysis was 12 months as this is the duration of the trial phase of the study. In this analysis, we adopted a health system/payer perspective. This narrower perspective stands in contrast to the full, societal perspective, which incorporates all relevant costs and benefits and is recommended for most economic evaluations [39]. While this narrower perspective can potentially ignore important costs or benefits from the broad societal standpoint, it has the practical value of explicitly addressing the budgetary concerns of payers. Thus, this approach fits well with implementation science contexts where financial factors are often central to whether programs and services are adopted and sustained [40].

Assumptions were made on the psychometric properties of the outcome measures, the effectiveness of the Life Goals intervention, and the reliability of time reporting by the facilitators. We test these assumptions in the sensitivity analyses by varying the costs and outcomes related to each intervention condition at low and high values (95% confidence interval). To address missing data on our utility (outcome) measures, we employed an inverse probability weighting (IPW) approach [41].

We estimated per-patient costs and QALYs for each implementation strategy sequence. We calculated the per-patient cost by dividing the total costs per condition by the number of patients in each condition. To compare interventions, we divided net incremental costs (net increase in costs from REP+EF/IF versus REP+EF, for example) by incremental effectiveness (net increase in QALYs in REP+EF/IF versus REP+EF groups, for example) to calculate the incremental cost-effectiveness ratio for patient-level outcomes across the conditions. We conducted a one-way sensitivity analysis on all input parameters listed in Table 1 to create a tornado diagram using net monetary benefits (NMB). We used NMB as this facilitates multiple comparisons, as in the current study, and incremental cost-effectiveness ratios (ICERs) are less suitable with more than 2 comparators [42]. The sensitivity analysis evaluated how costs and incremental cost-effectiveness are affected by variations in key parameters [30]. When available, we based upper/lower bound estimates on the 95% confidence intervals. We also conducted a probabilistic sensitivity analysis (PSA). PSA characterizes uncertainty in all parameters simultaneously, reflecting the likelihood that each model parameter takes on a specific value and provides information on overall decision uncertainty based on parameter uncertainty [43]. We conducted 1000 model simulations to quantify the probability that the implementation strategy is cost-effective for a range of thresholds of willingness-to-pay [44]. We conducted a scenario analysis to evaluate results for longer analytic time horizons, from 2 to 10 years. In this additional analysis, the effects of the intervention were assumed to remain constant over time, consistent with values estimated during the final phase for each condition of the trial.

## Results

### Results of base case analysis

**Fig. 2:**
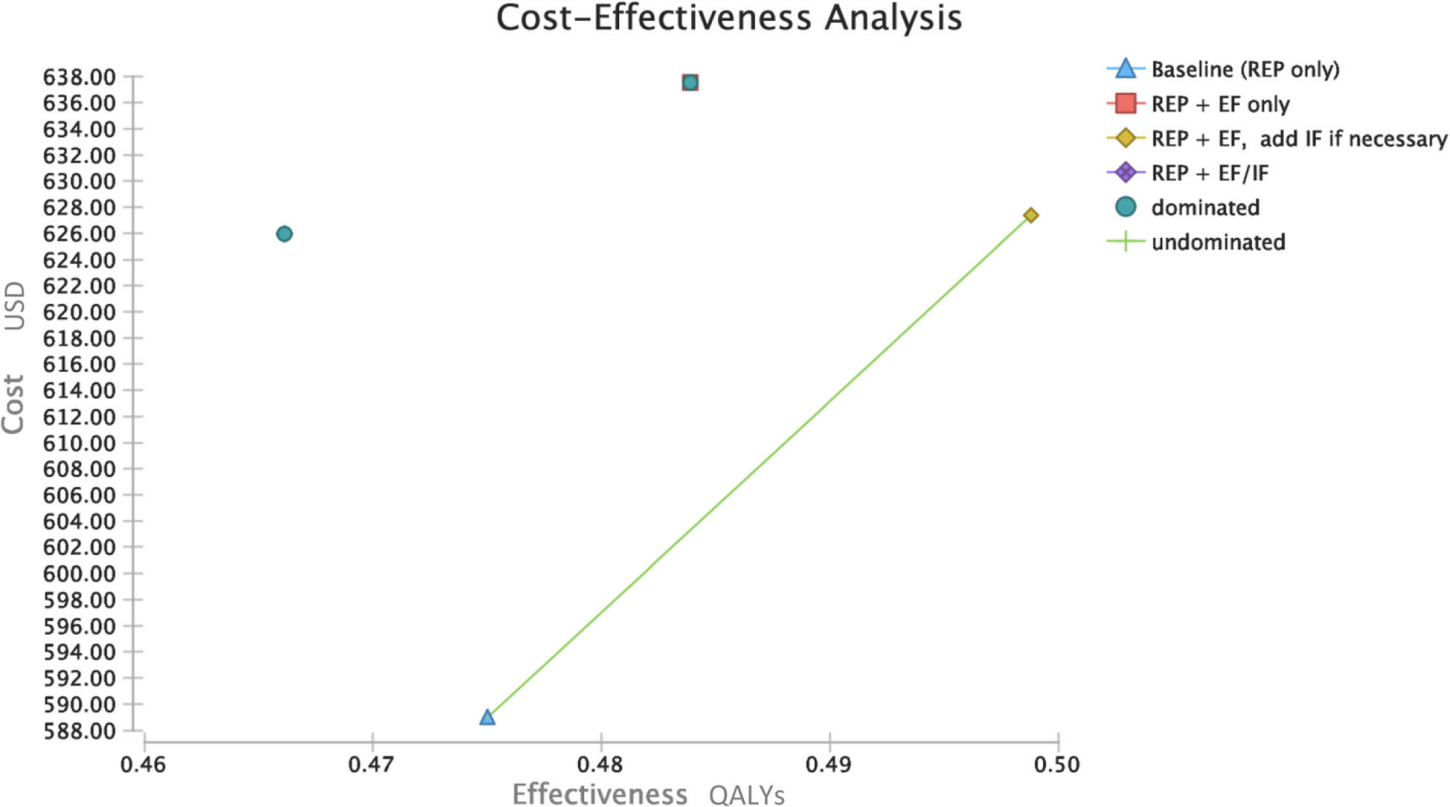
Cost-effectiveness plane, organization/payer perspective

Base case results are presented in Table 2, and a plot of cost-effectiveness across implementation strategies is depicted in Fig. 2. Our base case analysis results indicate REP ONLY is the least expensive. REP+EF, ADD IF has an ICER of $593/QALY. REP+EF had higher QALYs than REP alone, but the QALYs were not as high as REP+EF, ADD IF, and it was higher cost than REP+EF, ADD IF. REP+EF/IF had higher costs and lower QALYs than REP ONLY.

**Table 2:** Base case analysis results

Condition	Cost per patient	Effectiveness	ICER^**a**^	NMB^**b**^
**Utility: QALYS**				
Baseline (REP only)	588.95	0.47	0	46911.05
REP+EF only	637.53	0.48	Dominated^c^	47822.00
REP+EF, add IF if needed	627.40	0.54	593.42^d^	53351.18
REP+EF/IF	625.95	0.47	Dominated^c^	45987.68

^a^ICER: incremental cost-effectiveness ratio

^b^NMB: net monetary benefit. Willingness-to-pay threshold $100,000/QALY [45]

^c^Dominated: more expensive and less effective than comparators [46]

^d^Since “REP+EF/IF” is dominated, “REP+EF, add IF if needed” is compared to REP only

### Sensitivity analysis

**Fig. 3:**
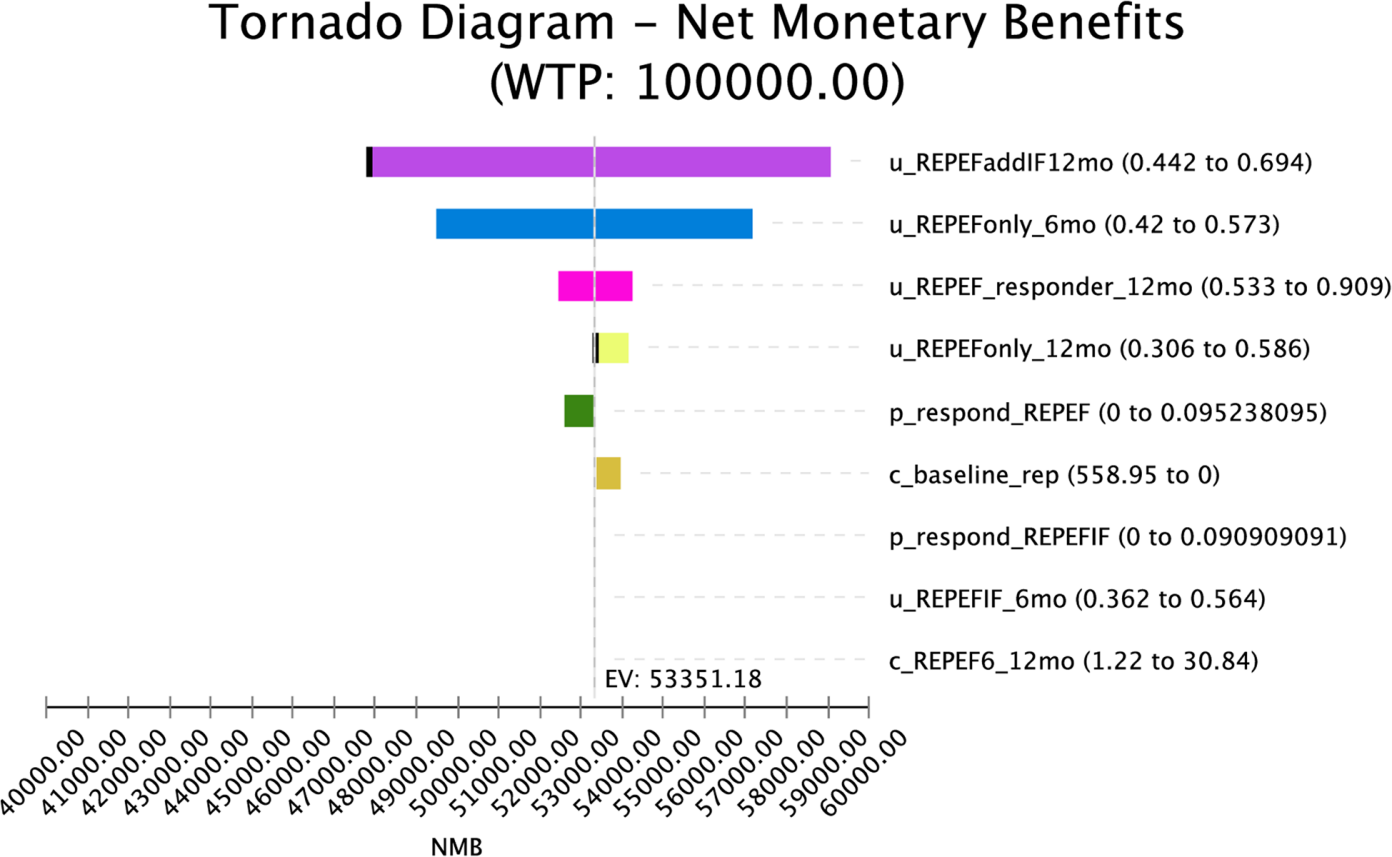
Tornado diagram showing one-way sensitivity analyses for the base case with the most sensitive parameters. All parameters were evaluated and data are provided in the appendix. Thick vertical black lines on the ends of the bars indicate values at which the initial preferred option is no longer cost-effective

**Fig. 4:**
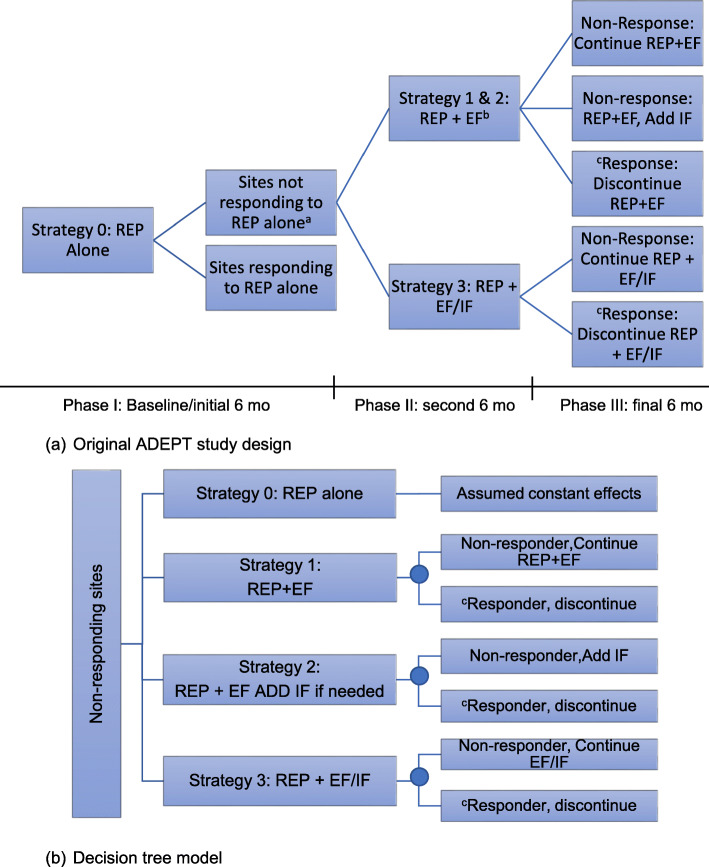
The original study design to evaluate effectiveness (a) and decision tree model to evaluate cost-effectiveness (b). This cost-effectiveness analysis focuses on implementation strategies for sites not responding to the REP alone intervention (the “sites not responding to REP alone” portion of the tree in 2a). In the original study, baseline data were gathered prior to initiation of the trial phase (Phase I). In this study, we sought to determine the most cost-effective option for deploying an implementation strategy with multiple components across its all possible permutations (e.g., REP+EF/IF) and comparing this to usual implementation (baseline REP). To accomplish this, we created the decision tree to represent all the decision options and their subsequent steps and estimate their respective costs and consequences to allow for comparison. This modeling approach represents the possible implementation strategy decision options for decision makers, quantifies the uncertainty, and allows for evaluation of alternatives. ^a^ In the original trial, non-responding sites were randomized following Phase I to REP+EF or REP+EF/IF. ^b^ Following Phase II, non-responding sites in the REP+EF condition were randomized again to either continue REP+EF or add IF (REP+EF/IF). Details of the trial are published elsewhere (see Kilbourne et. al., 2014). ^c^ Sites that responded to the implementation strategy after the initial 6 months of the Trial Phase: either < 10 patients receiving Life Goals or > 50% of patients receiving Life Goals had ≤ 3 sessions, min dose for clinically significant results. Sites that responded to the implementation strategy discontinued the strategy during the second 6 months/Phase III of the trial

To test our model assumptions, we conducted sensitivity analyses on all parameters (Appendix Table 3). In the tornado diagram (see Fig. 3), we found the results were most sensitive to the following variables (in order): utility of individuals in the REP+EF, ADD IF arm at Phase III, the utility of individuals in the REP+EF arm at Phase II, the utility of individuals in the REP+EF arm at Phase III for responders, and utility of individuals in the REP+EF only arm at Phase III. We then conducted threshold analyses for each of the most sensitive parameters. We found that at utility values below .44 for REP+EF, ADD IF at Phase III, the value of REP+EF, ADD IF is no longer cost-effective and REP+EF becomes the most cost-effective choice. We also found that at utility values above .57 for REP+EF at Phase III, REP+EF ADD IF is no longer the most cost-effective option and REP+EF becomes the most cost-effective choice.

In addition to the deterministic sensitivity analyses, we also conducted probabilistic sensitivity analysis. The results indicate that the intervention with the best results in terms of utility would be most preferred. The willingness-to-pay threshold was not important (unless using a $0 willingness-to-pay threshold). REP+EF, ADD IF is the optimal strategy in about 30% of iterations, REP ONLY is the optimal strategy in 31% of the iterations, and REP+EF/IF the optimal strategy in 22% of the iterations.

We also conducted sensitivity analyses to explore an extended time horizon. In this analysis, we investigated the effects of extending the utilities during the final 6-month period in each condition from the current 12-month time horizon to 10 years. We found that if there are no additional benefits beyond 1 year, our cost-effectiveness ratio of REP+EF, ADD IF is $593.42/QALY. If benefits continued to 2 years, the ICER was $223.06/QALY; at 3 years, the ICER was $137.34/QALY; and at 10 years, patients gain 1.14 QALYs and the cost-effectiveness ratio is $33.71/QALY. Full results are provided in Appendix Table 4. REP+EF, ADD IF remained the most cost-effective option with an extended time horizon.

## Discussion

Effective implementation of EBPs for mental health treatment in communities is critical to improving the public’s health. Most individuals suffering from depression and other mental health conditions are not receiving evidence-based practices (EBPs) such as Life Goals (LG) in community settings, resulting in poor and costly health outcomes and millions of research dollars wasted when EBPs fail to reach those most in need [10–12]. Implementation strategies are key to improving uptake of EBPs in communities and achieving public health objectives of evidence-based treatments such as Life Goals. Implementation strategies, however, vary in intensity and cost. More research is needed on applying these strategies with consideration of the economic impact, given that community clinics often have limited—and carefully allocated—resources to promote EBP uptake [47]. This research is vital to bridging the research-to-practice gap, but economic evaluation of implementation strategies remains understudied [47]. This study is one of the first to investigate the cost-effectiveness of implementation strategies as part of an adaptive trial. Adaptive trials are an effective way to accelerate research-to-practice translation by simultaneously evaluating multiple strategies and combinations of strategies, based on clinics’ needs.

We found that, overall, REP+facilitation in its various permutations is a relatively low-cost implementation strategy. Identifying the costs and potential utilities for each REP+facilitation combination can help decision makers with resource allocation for implementation. Understanding the resources needed to achieve desired behavioral outcomes (e.g., reduced ATOD use) is essential to implementing and sustaining EBIs [23]. Also, we found that REP+EF, ADD IF may be the most cost-effective implementation strategy. But these results are still uncertain based on highly variable quality-of-life assessments by participants. Although researchers have debated if a step-up versus step-down approach to evidence-based clinical treatment is most effective, the optimal approach for implementation strategies to enhance the uptake of these treatments is also unclear. Our results are consistent with other clinical research that suggests a step-up strategy is a more cost-effective approach [48]. This information will support organizations in making informed decisions by providing evidence that supports (or refutes) investment in specific implementation strategies as an efficient use of resources and thus can help prioritize implementation efforts.

We also found that stepping up non-responsive sites immediately to REP+EF/IF, the most intensive and costly strategy (at the site level), was not cost-effective. This may be for several reasons. First, EF alone may be sufficient for community clinics to effectively implement the Life Goals intervention and, thus, in most cases IF may not be necessary [31]. Second, many sites had difficulty identifying an internal facilitator. Subsequent analyses into time data indicate that the mean time to identify an IF was 69 days. This suggests that many sites assigned to the IF condition did not have one for the first 2 months of the evaluation period. These results also indicate that community clinics may have a limited capacity to identify and effectively utilize an IF. Finally, we may have had more favorable results with the REP+EF, ADD IF condition during Phase II as the EF was able to work with the clinic on their barriers to uptake immediately and may have been working with several versus a single staff member.

Our results were highly dependent on the assessment of utility. The utilities were variable and uncertain across the different intervention arms. This had a strong influence on the overall assessment of cost-effectiveness. Further research is needed that incorporates robust and relevant utilities in implementation research to identify the most cost-effective strategies. Although the trial only evaluated patients up until 1 year, our results did not change if we simulated a longer time horizon of benefits. Extending benefits out from the current trial (12 months) to 10 years, the cost-effectiveness of REP+EF, ADD IF improved to $33.71/QALY. As the clinical benefit from engaging in evidence-based practices for mental health treatment may extend beyond the time horizon of the trial itself, studies that only observe outcomes over a short time horizon may report artificially high CE ratios [49]. We have found that extending the time horizon does reduce the CE ratio.

### Limitations

We adopted a health payer perspective, which may not account for other relevant costs if considering the societal perspective. This may include indirect costs such as patient time, costs of hospitalization, or other treatments or lost productivity. Yet, this narrower perspective has the practical value of explicitly addressing the budgetary concerns of payers and fits well with implementation science contexts where financial factors are often central to whether programs and services are adopted and sustained [40]. We did not have additional information on estimates of REP costs to vary parameters and these cost estimates primarily relied on research team recall. There may be additional costs not included in the estimates that may have implications on the CEA results. Also, additional information to vary specific parameters may also help inform those parameters that are most influential on our estimates. In our CEA analyses, however, all groups had REP costs incorporated into total costs, so this is unlikely to influence the CEA results across the REP+facilitation permutations. We did not have a direct measure of QALYs and thus our utility estimates may be especially susceptible to measurement error. A notable amount of research has been done, however, on mapping the SF-12 components to the EQ-5D thus reducing the likelihood of error as a result of the mapping process. Next, we had a notable amount of missing patient-level survey data, increasing the likelihood of biased estimates. We did, however, attempt to reduce this bias using inverse probability weighting.

The current study would benefit from a mixed methods approach, specifically a sequential design, to obtain qualitative data following the quantitative data collection to better understand challenges to utilizing IF/EF. Finally, our study has a limited time horizon. Using incremental QALYs gained based on the survey results and running sensitivity analysis to evaluate potential effects of a longer time horizon showed REP+EF, ADD IF was still highly cost-effective. However, a longer time horizon within the RCT would provide additional information for a longer-term return-on-investment and could provide more confidence about which adaptive implementation strategy is best.

## Conclusions

Our study has several practice implications. First, our results support using a step-up strategy for implementation support for sites that are slow to implement as a cost-effective approach to enhancing uptake and clinical outcomes. Second, our results provide information for decision makers and community health clinic leadership on the costs and relative benefits of using various implementation strategies to improve clinical outcomes. Third, our results support the need for further cost-effectiveness research and incorporating robust utility assessments in community health clinics to provide evidence that will support or refute investments in specific strategies. Finally, our results point to the need for mid-program utility evaluation for both effectiveness and cost-effectiveness to make accurate determinations of the most efficient implementation strategy approach.

## Data Availability

Deidentified data are available on request.

## Appendix

**Table 3:** Tornado text report including results for all model input parameters

	Variable Low	Variable Base	Variable High	Impact	Low	High	Spread	Spread^2^	Risk %	Cum Risk %	Spread
Utility REPEFaddIF 12mo	0.442	0.568	0.694	Increase	47821.99524	59051.17619	11229.18095	126094504.9	0.666061436	0.666061436	11229.18095
Utility REPEFonly 6mo	0.42	0.497	0.573	Increase	49501.17619	57151.17619	7650	58522500	0.30912989	0.975191325	7650
Utility REPEF responder 12mo	0.533	0.721	0.909	Increase	52455.9381	54246.41429	1790.47619	3205804.989	0.016933831	0.992125157	1790.47619
Utility REPEFonly 12mo	0.306	0.446	0.586	Increase	53351.17619	54155.32857	804.152381	646661.0518	0.003415819	0.995540976	804.152381
Probability respond REPEF	0	0.095238095	0.095238095	Increase	52622	53351.17619	729.1761905	531697.9168	0.002808556	0.998349532	729.1761905
Cost baseline rep	0	588.95	558.95	Decrease	53381.17619	53940.12619	558.95	312425.1025	0.001650304	0.999999836	558.95
Cost REPEFIF 12mo	3.15	6.35	9.27	Decrease	53348.53429	53354.07143	5.537142857	30.65995102	1.61953E−07	0.999999998	5.537142857
Cost REPEF 6mo	32.39	32.7	33.01	Decrease	53350.86619	53351.48619	0.62	0.3844	2.03049E 09	1	0.62
Probability respond REPEFIF	0	0.090909091	0.090909091	Increase	53351.17619	53351.17619	0	0	0	1	0
Utility REPEFIF 6mo	0.362	0.463	0.564	Increase	53351.17619	53351.17619	0	0	0	1	0
Cost REPEF 12mo	1.22	17.55	30.84	Increase	53351.17619	53351.17619	0	0	0	1	0
Utility REPEFIF 12mo	0.392	0.479	0.566	Increase	53351.17619	53351.17619	0	0	0	1	0
Utility REPbaseline	0.43	0.475	0.521	Increase	53351.17619	53351.17619	0	0	0	1	0
Utility REPEFIF responder 12mo	0.184	0.372	0.559	Increase	53351.17619	53351.17619	0	0	0	1	0
Cost REPEFIF 6mo	28.51	31.23	32.22	Increase	53351.17619	53351.17619	0	0	0	1	0

*SPREAD*^*2*^ spread value, squared

**Table 4:** One-way sensitivity analysis results for extended time horizon

Year	Strategy	Strategyindex	Cost	Incr cost^**a**^	Eff^**b**^	Incr eff^**c**^	ICER^**d**^	NMB^**e**^	C/E^**f**^	Dominance
0	Baseline(REP only)	2	588.95	0	0.475	0	0	46911.05	1239.894737	
0	REP+EF/IF	1	625.9527273	37.00272727	0.466136364	− 0.008863636	− 4174.666667	45987.68364	1342.853242	(Dominated)
0	REP+EF, addIF if needed	0	627.3952381	38.4452381	0.539785714	0.064785714	593.4215362	53351.17619	1162.304265	
0	REP+EF only	3	637.5285714	10.13333333	0.484595238	− 0.055190476	− 183.6065574	47821.99524	1315.589839	(Dominated)
1	Baseline(REP only)	2	588.95	0	0.95	0	0	94411.05	619.9473684	
1	REP+EF/IF	1	625.9527273	37.00272727	0.935409091	− 0.014590909	− 2536.012461	92914.95636	669.175373	(Dominated)
1	REP+EF, addIF if needed	0	627.3952381	38.4452381	1.122357143	0.172357143	223.0556707	111608.319	558.9978574	
1	REP+EF only	3	637.5285714	10.13333333	0.956785714	− 0.165571429	− 61.20218579	95041.04286	666.3232549	(Dominated)
2	Baseline(REP only)	2	588.95	0	1.425	0	0	141911.05	413.2982456	
2	REP+EF/IF	1	625.9527273	37.00272727	1.404681818	− 0.020318182	− 1821.163311	139842.2291	445.618872	(Dominated)
2	REP+EF, addIF if needed	0	627.3952381	38.4452381	1.704928571	0.279928571	137.3394573	169865.4619	367.9891631	
2	REP+EF only	3	637.5285714	10.13333333	1.42897619	− 0.275952381	− 36.72131148	142260.0905	446.1435926	(Dominated)
3	Baseline(REP only)	2	588.95	0	1.9	0	0	189411.05	309.9736842	
3	REP+EF/IF	1	625.9527273	37.00272727	1.873954545	− 0.026045455	− 1420.69808	186769.5018	334.0277003	(Dominated)
3	REP+EF, addIF if needed	0	627.3952381	38.4452381	2.2875	0.3875	99.21351767	228122.6048	274.2711423	
3	REP+EF only	3	637.5285714	10.13333333	1.901166667	− 0.386333333	− 26.2295082	189479.1381	335.3354457	(Dominated)
4	Baseline(REP only)	2	588.95	0	2.375	0	0	236911.05	247.9789474	
4	REP+EF/IF	1	625.9527273	37.00272727	2.343227273	− 0.031772727	− 1164.606581	233696.7745	267.1327423	(Dominated)
4	REP+EF, addIF if needed	0	627.3952381	38.4452381	2.870071429	0.495071429	77.6559419	286379.7476	218.5991721	
4	REP+EF only	3	637.5285714	10.13333333	2.373357143	− 0.496714286	− 20.4007286	236698.1857	268.6188943	(Dominated)
5	Baseline(REP only)	2	588.95	0	2.85	0	0	284411.05	206.6491228	
5	REP+EF/IF	1	625.9527273	37.00272727	2.8125	− 0.0375	− 986.7393939	280624.0473	222.5609697	(Dominated)
5	REP+EF, addIF if needed	0	627.3952381	38.4452381	3.452642857	0.602642857	63.79439769	344636.8905	181.7144906	
5	REP+EF only	3	637.5285714	10.13333333	2.845547619	− 0.607095238	− 16.69150522	283917.2333	224.0442462	(Dominated)
6	Baseline(REP only)	2	588.95	0	3.325	0	0	331911.05	177.1278195	
6	REP+EF/IF	1	625.9527273	37.00272727	3.281772727	− 0.043227273	− 856.0042061	327551.32	190.7361598	(Dominated)
6	REP+EF, addIF if needed	0	627.3952381	38.4452381	4.035214286	0.710214286	54.13188508	402894.0333	155.48003	
6	REP+EF only	3	637.5285714	10.13333333	3.317738095	− 0.71747619	− 14.12358134	331136.281	192.1575945	(Dominated)
7	Baseline(REP only)	2	588.95	0	3.8	0	0	379411.05	154.9868421	
7	REP+EF/IF	1	625.9527273	37.00272727	3.751045455	− 0.048954545	− 755.8588672	374478.5927	166.8742048	(Dominated)
7	REP+EF, addIF if needed	0	627.3952381	38.4452381	4.617785714	0.817785714	47.01138382	461151.1762	135.8649528	
7	REP+EF only	3	637.5285714	10.13333333	3.789928571	− 0.827857143	− 12.24043716	378355.3286	168.2165137	(Dominated)
8	Baseline(REP only)	2	588.95	0	4.275	0	0	426911.05	137.7660819	
8	REP+EF/IF	1	625.9527273	37.00272727	4.220318182	− 0.054681818	− 676.6916043	421405.8655	148.3188471	(Dominated)
8	REP+EF, addIF if needed	0	627.3952381	38.4452381	5.200357143	0.925357143	41.54637849	519408.319	120.6446444	
8	REP+EF only	3	637.5285714	10.13333333	4.262119048	− 0.938238095	− 10.80038573	425574.3762	149.5801887	(Dominated)
9	Baseline(REP only)	2	588.95	0	4.75	0	0	474411.05	123.9894737	
9	REP+EF/IF	1	625.9527273	37.00272727	4.689590909	− 0.060409091	− 612.5357412	468333.1382	133.477043	(Dominated)
9	REP+EF, addIF if needed	0	627.3952381	38.4452381	5.782928571	1.032928571	37.21964825	577665.4619	108.4909195	
9	REP+EF only	3	637.5285714	10.13333333	4.734309524	− 1.048619048	− 9.66350302	472793.4238	134.6613626	(Dominated)
10	Baseline(REP only)	2	588.95	0	5.225	0	0	521911.05	112.7177033	
10	REP+EF/IF	1	625.9527273	37.00272727	5.158863636	− 0.066136364	− 559.4914089	515260.4109	121.3353892	(Dominated)
10	REP+EF, addIF if needed	0	627.3952381	38.4452381	6.3655	1.1405	33.70910837	635922.6048	98.56181574	
10	REP+EF only	3	637.5285714	10.13333333	5.2065	− 1.159	− 8.743169399	520012.4714	122.4485876	(Dominated)

^a^Incremental cost

^b^Effectiveness

^cI^ncremental effectiveness

^d^Incremental cost-effectiveness ratio

^e^Net monetary benefit

^f^Cost-effectiveness

## Abbreviations

EBPs: Evidence-based practices
REP: Replicating Effective Programs
CDC: Centers for Disease Control and Prevention
iPARIHS: Integrating Promoting Action on Research Implementation in Health Services
IF: Internal facilitation/facilitator
EF: External facilitation/facilitator
VA: Veteran’s Administration
CEA: Cost-effectiveness analysis
ADEPT: Adaptive Implementation of Effective Programs Trial
SMART: Sequential multiple assignment randomized trial
LMSW: Licensed Masters of Social Work
QALYs: Quality-adjusted life years
IPW: Inverse probability weighting
NMB: Net monetary benefits
ICER: Incremental cost-effectiveness ratio
PSA: Probabilistic sensitivity analysis
